# Relationship between the area of isopters and Vigabatrin dosage during two years of observation

**DOI:** 10.1186/1471-2415-14-56

**Published:** 2014-04-30

**Authors:** Katarzyna Nowomiejska, Marian Jedrych, Agnieszka Brzozowska, Konrad Rejdak, Tomasz Zarnowski, Michael J Koss, Katarzyna Ksiazek, Piotr Ksiazek, Ryszard Maciejewski, Anselm G Juenemann, Ulrich Schiefer, Robert Rejdak

**Affiliations:** 1 Department of General Ophthalmology, Medical University, ul. Chmielna 1, 20-079 Lublin, Poland; 2 Department of Mathematics and Medical Biostatistics, Medical University, Lublin, Poland; 3 Department of Neurology, Medical University, Lublin, Poland; 4 Department of Glaucoma Diagnostics and Microsurgery, Medical University, Lublin, Poland; 5 Department of Ophthalmology, University of Heidelberg, Heidelberg, Germany; 6 Department of Public Health, Medical University, Lublin, Poland; 7 Human Anatomy Department, Medical University in Lublin, Lublin, Poland; 8 Department of Ophthalmology, University of Erlangen-Nürnberg, Erlangen, Germany; 9 Centre of Ophthalmology, Institute for Ophthalmic Research, University of Tuebingen, Tuebingen, Germany; 10 Department of Experimental Pharmacology, Medical Research Centre, Polish Academy of Sciences, Warsaw, Poland

**Keywords:** Vigabatrin-attributable visual field loss, Isopter area, Reaction time, Perimetry

## Abstract

**Background:**

The aim of the study was to evaluate the relationship between the area of isopters obtained using semi-automated kinetic perimetry (SKP) and Vigabatrin dosage in epilepsy patients with pretreatment baseline examination during 2-years of the follow-up.

**Methods:**

29 epilepsy patients were included into the study, but 15 individuals were excluded due to cognitive impairment, intracranial pathologies or eye diseases. Finally, 14 patients were examined with SKP before VGB treatment and after 6, 12, 18, and 24 months. Reaction time (RT)-corrected areas of three isopters (III4e, I4e and I2e) were measured for each of five examinations and compared intra-individually during 2-years period. Additionally, six epilepsy patients on other antiepileptic drugs were examined five times with SKP as a control.

**Results:**

There was a significant decrease of I2e, I4e and III4e isopters’ area during the follow-up of two years. Correlation was found between the I2e isopter’s area and both cumulative dose and mean daily dose of VGB. With increasing RT, there was decreasing of all isopters’ area in patients receiving VGB. In epilepsy patients who were not receiving VGB, there were no significance differences in isopters’ area during follow-up.

**Conclusion:**

There was attenuation of area of III4e, I4e and I2e isopters obtained with SKP during a period of 2 years. RT, the cumulative dose and the mean daily dose of VGB influenced isopters' area obtained with SKP.

## Background

Vigabatrin (VGB) (Sabril, Hoechst Marion Russel/Aventis Ltd) is a selective irreversible inhibitor of the gamma-aminobutric acid -transaminase [1] used in the management of partial epilepsy if it cannot be controlled satisfactorily by conventional therapy. Since 1997 many studies have reported symmetrical bilateral constriction of the visual field (VF) associated with VGB intake [2] with normal appearance of the optic nerve head and retina [3]. The incidence rate of VGB-induced concentric constriction of the VF varies with the method used to assess the VF, ranging from 17% [4] to 92% [5] of exposed adults.

Perimetry seems to be the best method to screen and monitor the visual function in patients taking VGB, however, there is no consensus in the literature as to the optimum perimetric test and there is no good method to quantify the VF loss. In clinical practice most of the patients treated with VGB are examined with static perimetry within 60° [6] or 30° [7] or 24° [8] of eccentricity. However, there is lack of validated scoring system for analysing supra-threshold screening programmes and unacceptable poor reliability indices have been observed while examining epilepsy patients with full threshold automated static perimeter [3,9]. Monitoring of visual function to understand the occurrence and manage the potential consequences of peripheral VF defects is now required for all patients who receive VGB [10].

Semi-automated kinetic perimetry (SKP) provides semi-automatic assessment of the entire (90˚) VF using Goldmann stimuli, which are presented along selected meridians with a constant angular velocity [11]. Results of SKP have been shown to be comparable to those of Goldmann manual kinetic perimetry [12] and static automated perimetry [13]. The major advantage of SKP is the standardisation of parameters and the ability to measure the isopters’ area in square degrees (deg^2^) and to consider reaction time (RT) in milliseconds (ms).

The aim of this prospective study was to evaluate the relationship between the area of isopters obtained using SKP and Vigabatrin dosage during a 2-years follow-up period in patients with pretreatment baseline examination.

## Methods

### Participants

The study was a prospective and observational case series. It was approved by the Ethics Committee of the Medical University in Lublin and performed in accordance with the ethical standards laid down in the 2008 Declaration of Helsinki.

A total of 29 adult patients with focal epilepsy and a clinical presentation of complex partial seizures with or without secondary generalisation (mean disease duration of 10 ± 4 years and mean seizure frequency of 5 ± 4/month at the study inclusion) were prospectively identified and selected by the neurologist from the Neurology Outpatient Clinic in Lublin and referred to the Department of Ophthalmology in Lublin. Inclusion criteria were as follows: at least 18 years of age, refractive errors of < 3 D sphere and < 1 D cylinder, transparent ocular media, pupil diameter more than 3 mm. The exclusion criteria were symptoms or signs of diseases other than epilepsy, in particular relating to visual function and patients who had had prior treatment that could have affected visual function. Overall 15 patients of the cohort were excluded. Nine patients were excluded due to cognitive deficiency. Two patients were excluded due to the ophthalmological abnormalities (optic nerve head drusen and cataract) and four patients - due to the intracranial pathology (encephalitis, cerebral palsy and stroke) causing hemianopic defects. Consequently, 14 patients (8 females, 6 males) remained in the study. The details of the demographic data (age, gender, visual acuity), drug history (mean daily dose, cumulative dose, duration of VGB treatment, duration of the follow-up, other antiepileptic drugs) and classification of VF results of each patient are shown in a table (Table 1). Additionally, six patients never exposed to VGB treated with other antiepileptic drugs were examined five times as a control group. Median visual acuity of this group was 1.0, mean age was 38 years (range 26–51 years).

**Table 1:** Demographic data and drug history of each of 14 patients taking Vigabatrin

**No**	**Age**	**Gender**	**Visual acuity**		**Mean daily dose of VGB (mg)**	**Cumulative dose of VGB (g)**	**Duration of VGB treatment (months)**	**Duration of follow-up (months)**	**Other antiepileptic drugs**	**Classification of the SKP visual field**
			**Right**	**Left**						
1	54	M	1.0	0.9	1800	1470	27	25	OCZ	Normal
2	23	F	1.0	1.0	1450	1050	24	24	VPA, LTG	Normal
3	41	F	1.0	1.0	1000	1230	21	24	OCZ	Mildly abnormal
4	53	F	1.0	1.0	1000	750	23	25	VPA	Normal
5	48	F	1.0	1.0	1500	1305	29	24	CBZ, CLZ	Normal
6	34	M	1.0	1.0	2100	1440	23	23	VPA, CBZ	Normal
7	27	M	1.0	1.0	1900	1710	29	25	VPA, CBZ	Normal
8	56	F	1.0	1.0	1000	1290	27	21	CBZ	Normal
9	59	M	0.7	1.0	2300	1365	24	24	VPA	Mildly abnormal
10	49	F	1.0	1.0	2000	1740	24	21	CBZ	Normal
11	48	M	1.0	1.0	1500	11305	24	22	CBZ	Normal
12	31	F	1.0	1.0	1000	300	11	14	CBZ	Normal
13	39	F	1.0	1.0	1000	6000	15	12	CBZ	Normal
14	35	M	1.0	1.0	1000	750	25	25	CBZ	Normal

F-female, M-male.

VGB-Vigabatrin.

mg-miligram.

g-gram.

CBZ – carbamazepin.

CLZ – clonazepam.

OCZ – oxcarbazepin.

VPA- valproate.

LTG-lamotrigine.

TGB-tiagabine.

TPM-topiramate.

SKP-semi-automated kinetic perimetry.

Patients had to have the diagnosis of epilepsy confirmed, according to the recommendations of the International League Against Epilepsy [14]. All patients underwent a standard blood analysis (liver and renal function, serum electrolytes, basic haematological indices) performed not longer than 1 week prior to the enrollment. All patients were carefully interviewed and clinically examined by a neurologist. The seizure frequency was monitored and calculated based on the available diaries provided by patients. Disease duration was estimated by determining the time from the first reported seizure. The mean age of patients at the time of inclusion was 42 years (range 23–59 years). On initial testing written informed consent was taken from all participants after explaining the nature of the study. All patients underwent a full ophthalmological examination including visual acuity testing, slit lamp examination, applanation tonometry and fundus examination. The mean of the best-corrected visual acuity of right eye was 0.97 (range 0.7-1.0) and 0.99 (range 0.9-1.0) of the left eye. The visual acuity remained stable during consecutive visits. None of the patients reported VF constriction.

### Medication

All patients received VGB as an adjunctive treatment to other antiepileptic drugs according clinical indications in order to increase seizure control. They all responded with good seizure control and gave consent to continue the treatment, being aware of potential side effects relating to visual function. Patients were examined first before starting treatment with VGB. The mean of the daily dose of VGB was 1535 mg/d (range 1000–2300 mg/d). At the time of the study, VGB was combined with other antiepileptic drugs: carbamazepin (CBZ) (64%), clonazepam (CLZ) (7%), oxcarbazepin (OCZ) (14%), valproid acid (VPA) (35%), lamotriginum (LTG) (7%). Patients from the control group received CBZ 67%, CLZ (33%) or gabapentinum (17%).

The mean duration of VGB therapy was 23 months (range 11–25 months). To calculate the cumulative dose (in grams) the daily dose was multiplied by the duration of therapy and then divided by 1000. The mean cumulative dose of VGB was 1164 g (range 300–1740 g) in this group.

### VF examination and analysis

VF examinations were performed with the SKP implemented in Octopus 101 instrument (Haag-Streit Inc., Koeniz, Switzerland). The same examiner (KN) performed all VF examinations. Both eyes were tested in all patients with the right eye tested before the left. The patients had no previous experience in the VF examination. Fourteen patients were examined five times: at baseline (i.e. before treatment) and 6, 12, 18, and 24 months after VGB intake. The mean examination duration was 11 minutes for both eyes (range 6–16 minutes). For the control group the examination duration was 12 min for both eyes (range 9–15 min).

During the VF examination the patient was instructed to look at the fixation target and push the button as quickly as possible when perceiving the stimulus. Stimuli V4e, III4e and I4e according to the Goldmann classification moving with the constant angular velocity of 3 degrees per second were used. The blind spot was assessed using I4e stimulus. RT was assessed by vectors located within intact areas of III4e isopter – two in the centre and two in the periphery of the VF. The periphery of the VF was examined without correction, an appropriate near correction was used for the central 30°. Fixation was monitored by the examiner using the video display of the instrument. The area of each isopter was corrected for RT and measured automatically in deg^2^ after each examination. The areas of three isopters (III4e, I4e and I2e) were measured for each of five examinations and compared intra-individually for each patient.

VF constriction was assessed by two observers, masked to the patients’ clinical status, according to the classification proposed by Wild and Kälviäinen [15,16] and used by Vanthalo [17] This classification is based on the temporal region of the VF. Three stages were described: (I) normal VF - extending out of 70˚ in the temporal meridian, (II) mildly abnormal - between 70˚ and 50˚ of the temporal meridian, (III) severely abnormal – less than 50˚ of the temporal meridian.

### Statistical analysis

As both eyes of each patient were examined, the area of isopters and RT of right eyes and left eyes were analyzed separately. Shapiro-Wilk test showed that the distribution of the data is not normal, nonparametric test Friedman Chi^2^ ANOVA was used for multiple comparisons of each isopter area and RT during the follow-up. As post-hoc analysis nonparametric Wilcoxon test was used to show differences in isopters’ area between visits. The Spearman correlation was assessed between the VF loss and age, RT, the mean daily dose, and cumulative dose of VGB. Statistical computations were performed using STATISTICA 10.0 (StatSoft, Poland) software.

## Results

**Figure 1:**
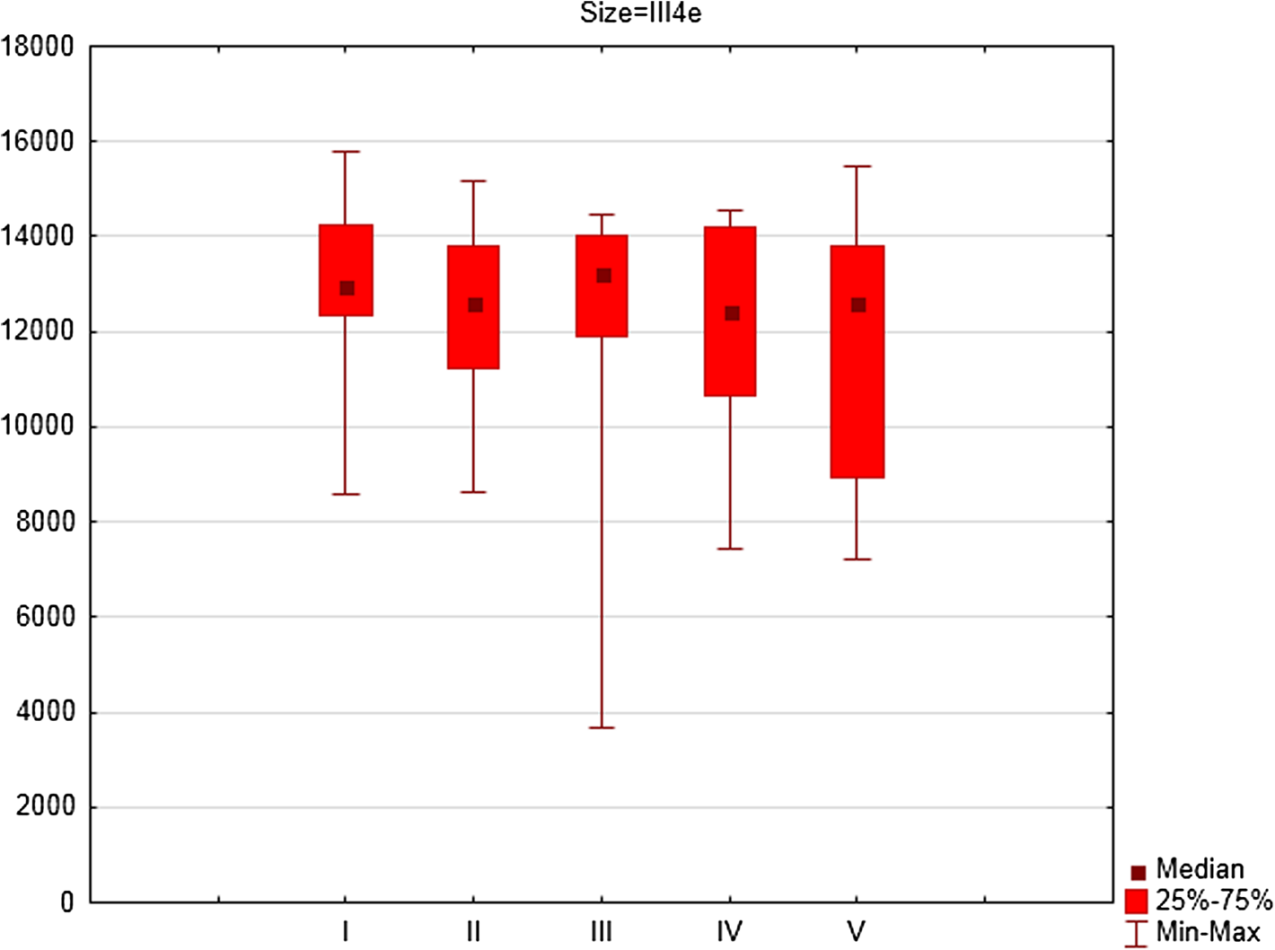
The area of III4e isopter in square degrees (medians, 25 and 75% quartiles and maximum and minimum values) of right eyes of patients on Vigabatrin treatment during 2- years follow-up period (five consecutive SKP examinations).

**Figure 2:**
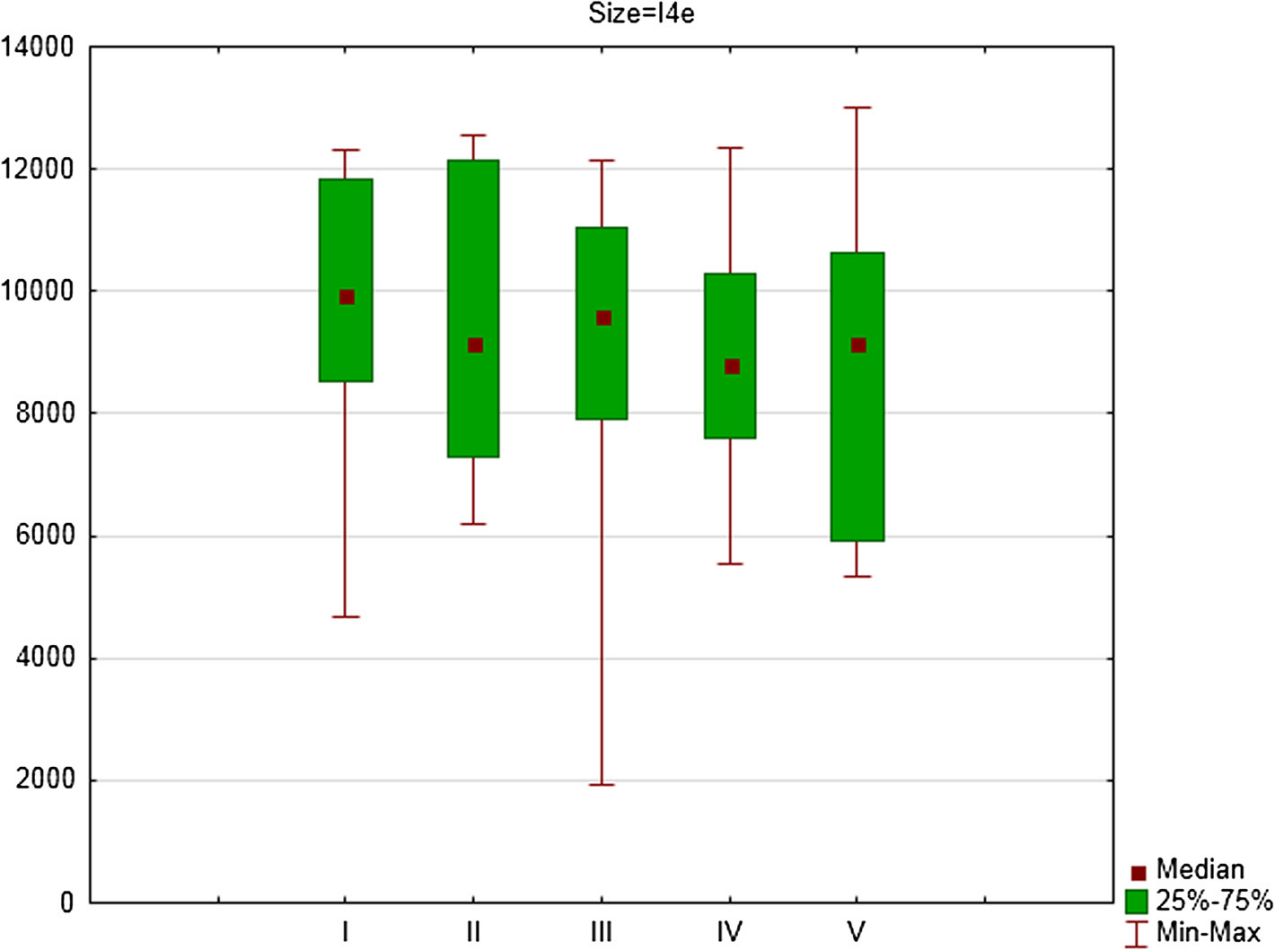
The area of I4e isopter in square degrees (medians, 25 and 75% quartiles and maximum and minimum values) of right eyes of patients on Vigabatrin treatment during 2- years follow-up period (five consecutive SKP examinations).

**Figure 3:**
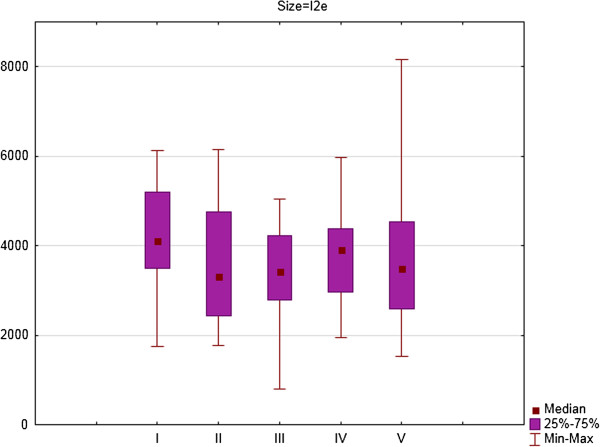
The area of I2e isopter in square degrees (medians, 25 and 75% quartiles and maximum and minimum values) of right eyes of patients on Vigabatrin treatment during 2- years follow-up period (five consecutive SKP examinations).

For the right eye there were significant differences between consecutive examinations for area of isopters: III4e (p = 0.031) (Figure 1), I4e (p = 0.003) (Figure 2) and I2e (p = 0.022) (Figure 3). For the right eye the mean area of III4e isopter was 13145.34 deg^2^ (standard deviation [SD] 1695.16 deg^2^) during the first examination and 11917.47 deg^2^ (SD 2590.82 deg^2^) during the fifth examination (Figure 1). The mean area of I4e isopter was 9863.88 deg^2^ (SD 1874.24 deg^2^) during the first examination and 8795.90 deg^2^ (SD 2522.65 deg^2^) during the fifth examination (Figure 2). The mean area of I2e isopter was 4120.17 deg^2^ (SD 1244.25 deg^2^) during the first examination and 3829,05 deg^2^ (SD 1804.21 deg^2^) during the fifth examination (Figure 3).

For the right eye there were significant differences in area of III4e isopter between the first and second examination (Z = 1.98; p = 0.05) and the first and fourth examination (Z = 2.73; p = 0.006). For I4e isopter the differences were significant between the first and third examination (Z = 2.73; p = 0.006) and between the first and fourth examination (Z = 2.54; p = 0.01). For I2e isopter the differences were significant between the first and third examination (Z = 2.67; p = 0.008) and between the first and fourth examination (Z = 2.23; p = 0.03).

**Figure 4:**
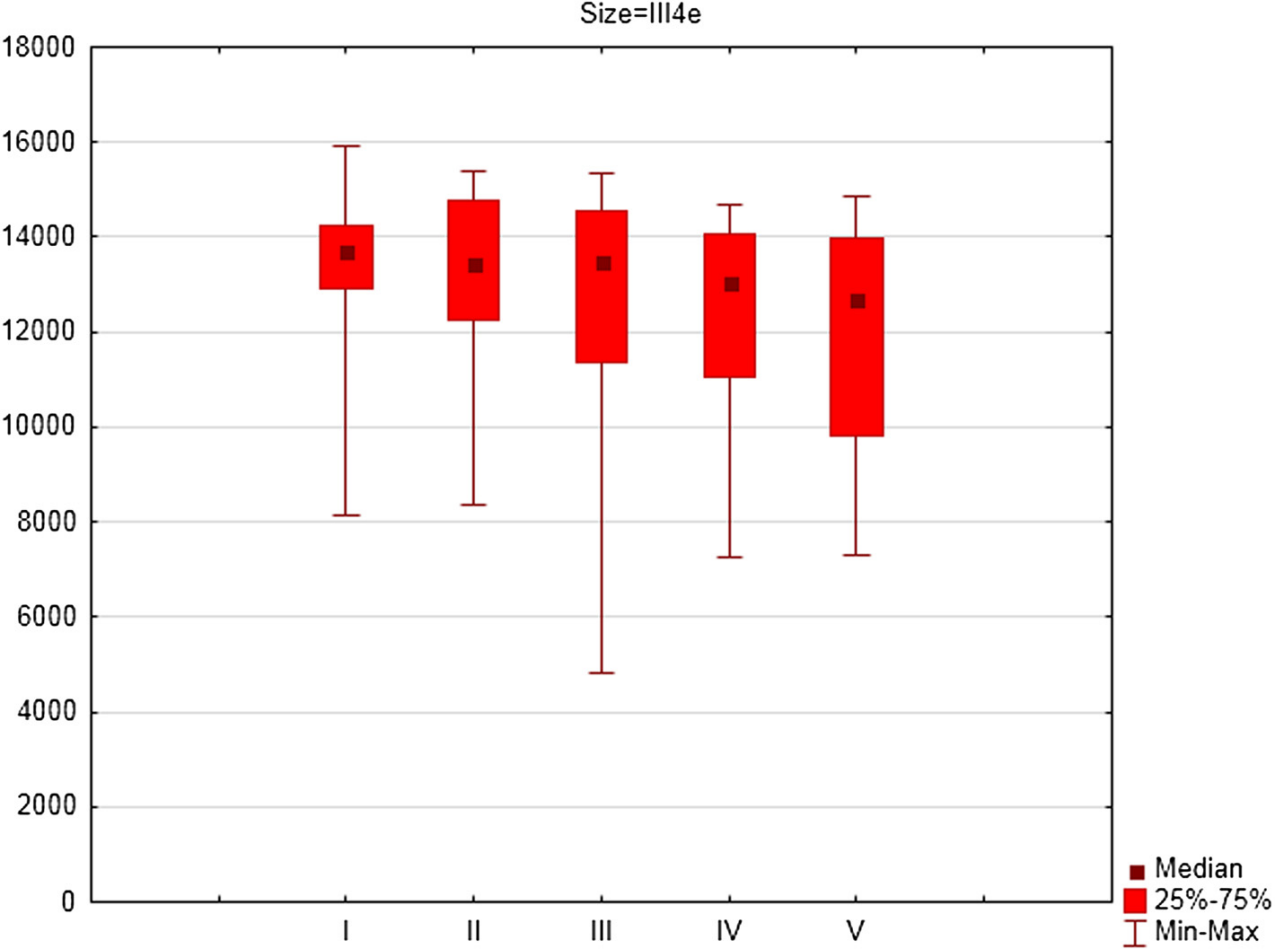
The area of III4e isopter in square degrees (medians, 25 and 75% quartiles and maximum and minimum values) of left eyes of patients on Vigabatrin treatment during 2- years follow-up period (five consecutive SKP examinations).

**Figure 5:**
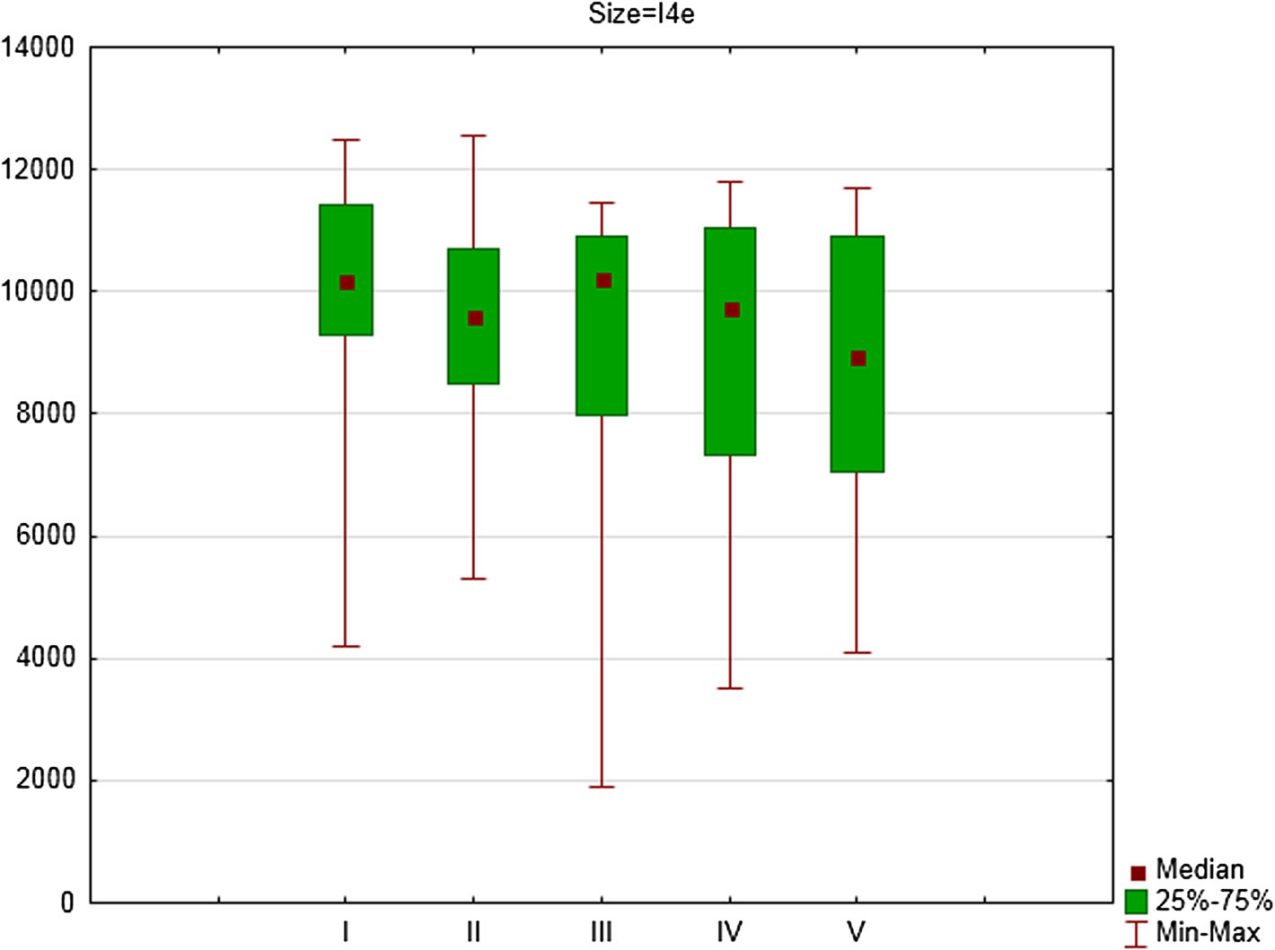
The area of I4e isopter in square degrees (medians, 25 and 75% quartiles and maximum and minimum values) of left eyes of patients on Vigabatrin treatment during 2- years follow-up period (five consecutive SKP examinations).

**Figure 6:**
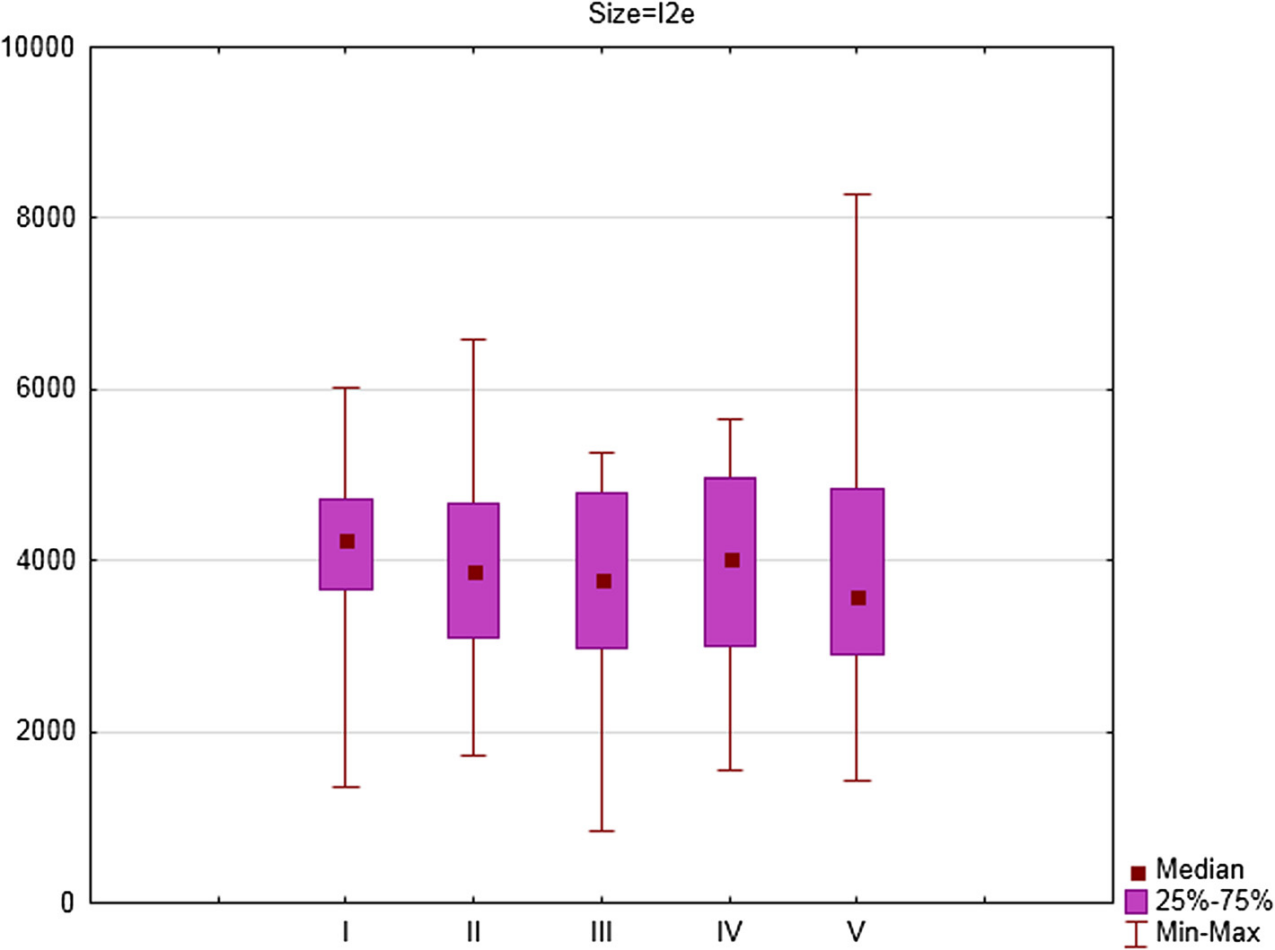
The area of I2e isopter in square degrees (medians, 25 and 75% quartiles and maximum and minimum values) of left eyes of patients on Vigabatrin treatment during 2- years follow-up period (five consecutive SKP examinations).

For the left eye, there were also significant differences between consecutive examinations for III4e (p = 0.001) (Figure 4), I4e (p = 0.044) (Figure 5) and I2e (p = 0.008) (Figure 6) isopters. For the left eye the mean area of III4e isopter was 13445.71 deg^2^ (SD 1575.41 deg^2^) during the first examination and 12161.48 deg^2^ (SD 2314.69 deg^2^) during the fifth examination (Figure 4). The mean area of I4e isopter was 10217.37 deg^2^ (SD 1762.10 deg^2^) during the first examination and 8841,68 deg^2^ (SD 2331.54 deg^2^) during the fifth examination (Figure 5). The mean area of I2e isopter was 4152.29 deg^2^ (SD 1044.73 deg^2^) during the first examination and 3853.79 deg^2^ (SD 1552.76 deg^2^) during the fifth examination (Figure 6).

For the left eye there were significant differences in area of III4e isopter between the first and fourth examination (Z = 2.42; p = 0.02) and the first and fifth examination (Z = 2.10; p = 0.04). For I4e isopter the differences were significant between the second and fourth examination (Z = 2.54; p = 0.01) and between the second and fifth examination (Z = 2.10; p = 0.04). For I2e isopter the differences were significant differences between the third and fourth examination (Z = 2.79; p = 0.005).

Hence, the area of all isopters decreased significantly between 5 examinations during 2-years follow-up both for the right and left eye.

In epilepsy patients who were not receiving VGB, there were no significance differences (p > 0.05) in III4e, I4e and I2e isopters’ area between examinations during follow-up period.

Correlation was found between the area of all isopters and individual RT for III4e R = −0.69; p = 0.002), for I4e (R = −0.57; p = 0.004),for I2e (R = −0.43; p = 0.004) for the right eye and for III4e (R = −0. 61; p = 0.002), I4e (R = −0.52; p = 0.017) and I2e (R = −0.49; p = 0.024) for the left eye.

Correlation was found between the area of isopters and cumulative dose of VGB for I2e isopter for the right eye (R = −0.63; p = 0.001) and I2e for the left eye (R = −0.46; p = 0.031).

Correlation was also found between the isopters’ area and the mean daily dose of VGB for I4e (R = −0.49; p = 0.02) and I2e (R = −0.78; p = 0.00001) for the right eye and for III4e (R = −0.42; p = 0.042) and I2e (R = −0.73; p = 0.008) for the left eye. Thus, with increasing cumulative dose of VGB there is decreasing of area of I2e isopter for both eyes. With increasing mean daily dose of VGB there is decreasing of I4e and I2e isopters’ area for the right eye and decreasing of III4e and I2e isopters’ area for the left eye.

There was no correlation found between isopters’ area and age of patients for the right eye (III4e p = 0.332, I4e p = 0.107 and I2e p = 0.617) and for the left eye (III4e p = 0.998, I4e p = 0.324 and I2e p = 0.461).

**Figure 7:**
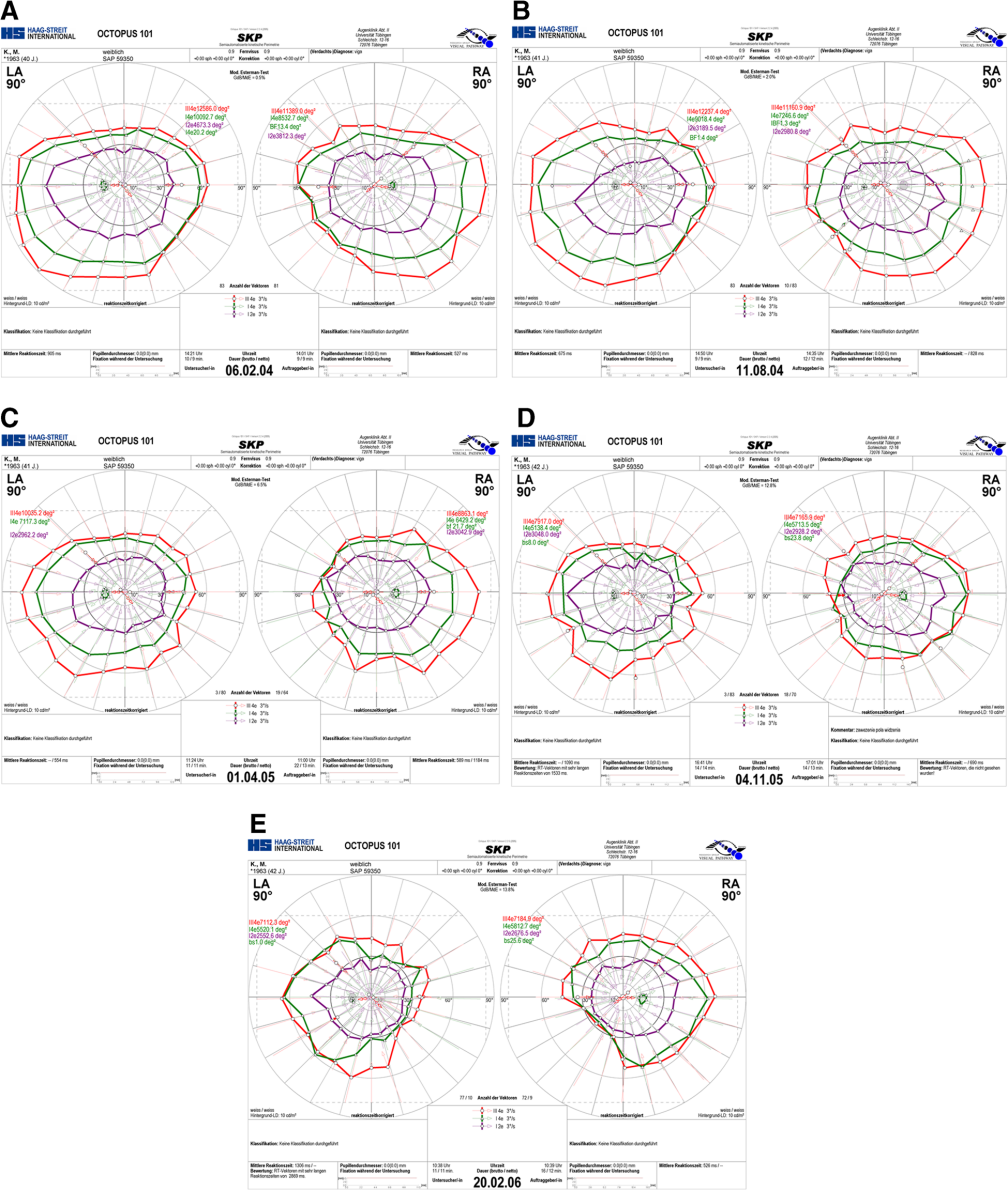
**Results of five consecutive examinations of semi-automated kinetic perimetry (Octopus 101) of a patient with Vigabatrin-attributable visual field loss.** Mean daily dose 1950 mg, cumulative dose 1230 g, duration of VGB treatment - 24 months. Other antiepileptic drug: oxcarbazepin.

After careful individual qualitative analysis of each patient VF series by two masked graders, the VFs of 2 of 14 patients (14%) - patient number 3 and number 9 - were classified as VAVFL (Figure 7a-d). The VF constriction was mild in the temporal meridian according to the classification of Wild and Kalvainen, with a depression observed nasally. VGB was discontinued in both two patients who developed VAVFL.

**Figure 8:**
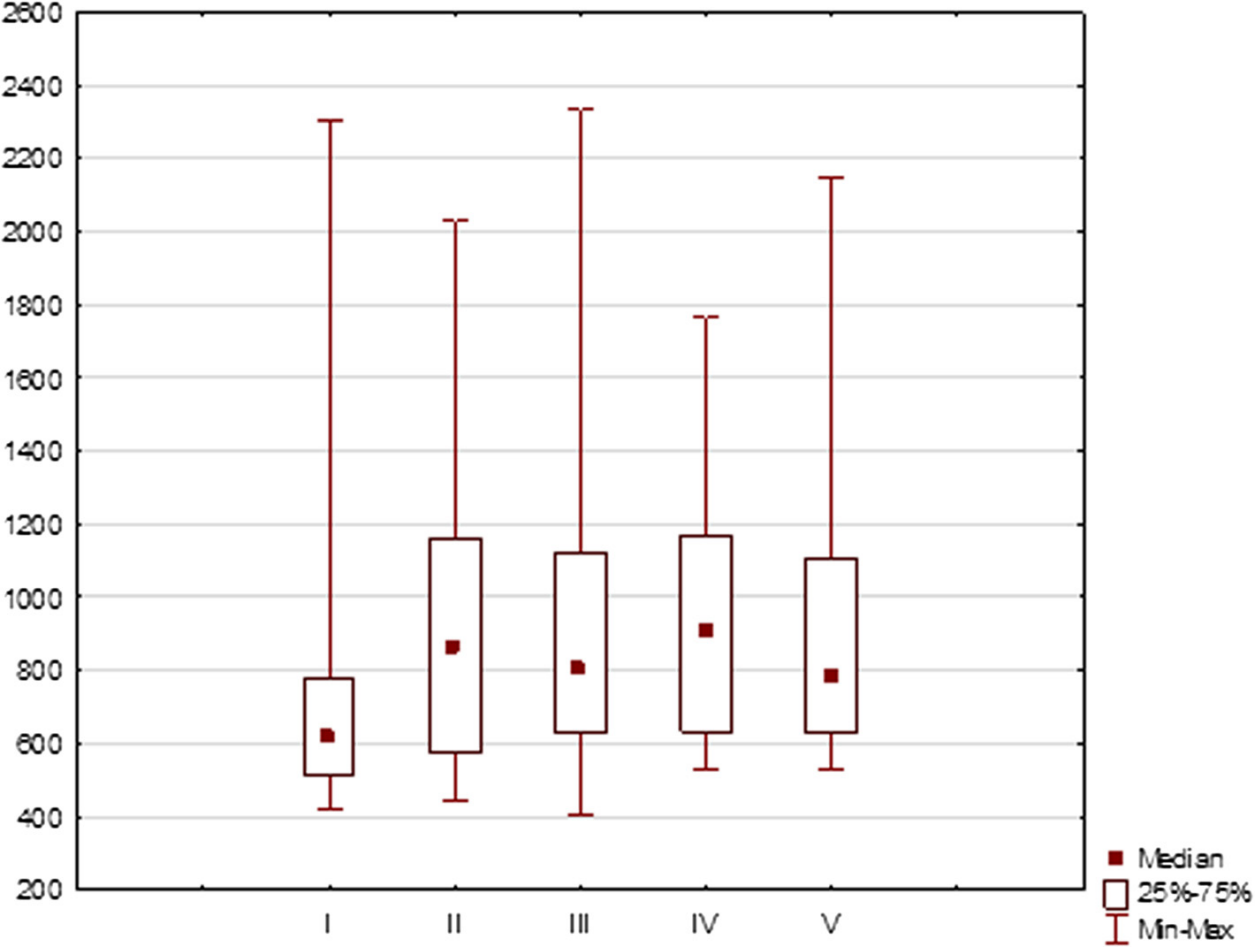
Reaction time values in milliseconds (medians, 25 and 75% quartiles, maximum and minimum values) of right eyes during five SKP examinations of patients taking Vigabatrin during 2-years period.

**Figure 9:**
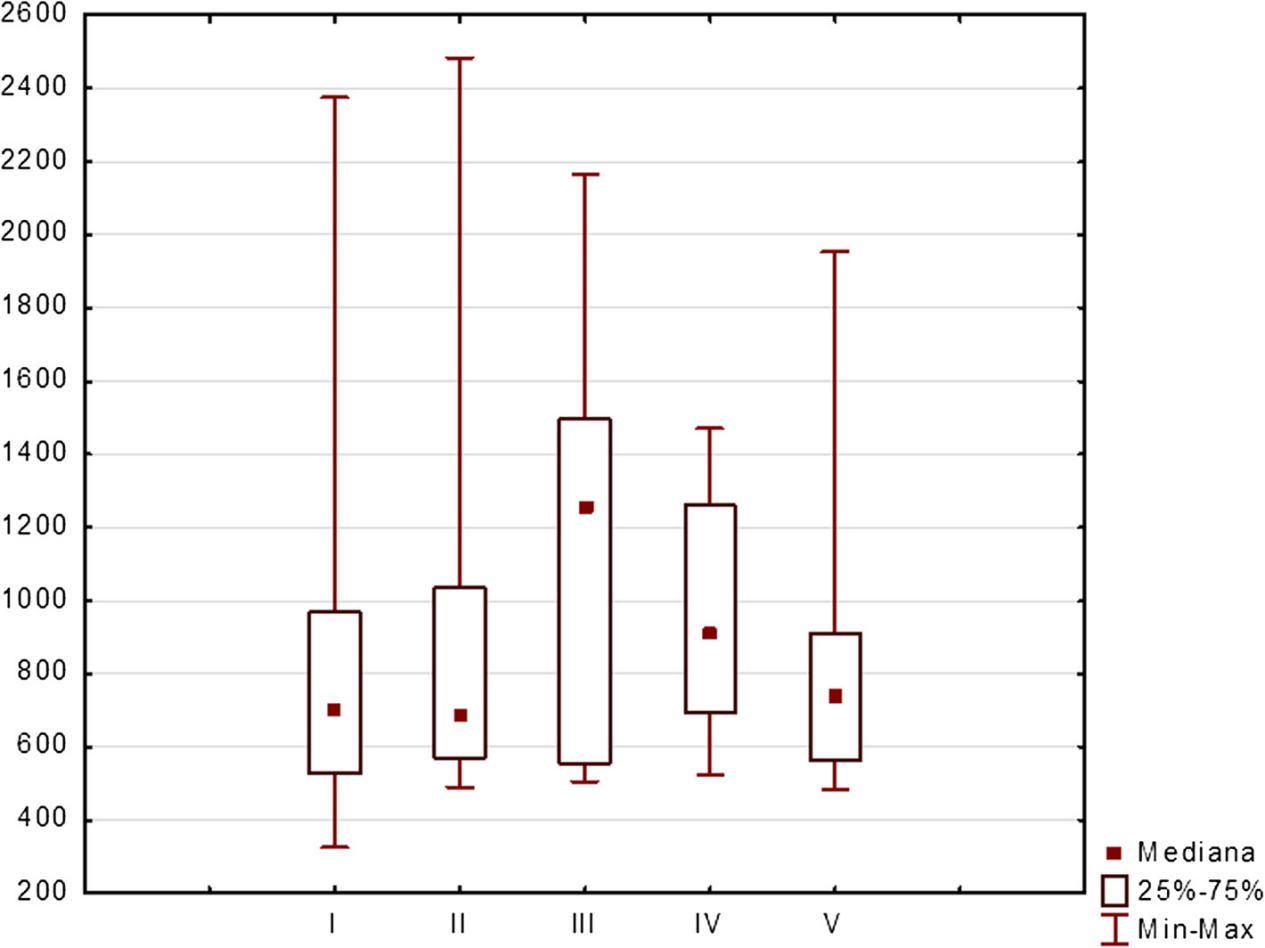
Reaction time values in milliseconds (medians, 25 and 75% quartiles, maximum and minimum values) of left eyes during five SKP examinations of patients taking Vigabatrin during 2-years period.

The mean RT was 940.9 ms (range 310–2500 ms) for the whole group. The differences in RT between examinations were not significant for the right eye (p = 0.400) (Figure 8) and for the left eye (p = 0.111) (Figure 9).

There was a correlation found between RT and area of III 4e (R = −0,46) , I4e (R = −0,52) and I 2e (R = −0,52) isopters. Thus, the longer RT, the smaller isopter’s area.

## Discussion

This is the first prospective investigation dealing with repeated SKP examinations over a 2-years follow-up in patients undergoing VGB therapy with the baseline examination performed before therapy.

There is lack of adequate prospective studies with VGB pre-treatment baseline examinations in the literature. Thus, the natural history of the VF loss is not known. The guidelines regarding monitoring VGB patients published by the Royal College of Ophthalmologists recommended pretreatment baseline VF examination and follow-up with VF examinations every 6 months during first three years of treatment [10]. However, 1/2 of the individuals who were initially screened for our study were not able to perform perimetry due to cognitive impairment. It is already known that approximately 20% patients exposed to VGB are unable to perform perimetry [18]. In the study of Kinirions [19] 152 patients were initially identified, but finally 93 were analysed. Forty-six patients were unsuitable for VF assessment because of moderate or severe learning disability and 11 patients had VFs that were thought to be unreliable.

Moreover, structural measures (particularly ocular coherence tomography - OCT) might also be obtained in these patients instead of VF testing, as Wild [20] and other authors [21] found attenuation of peripapillary RNFL in patients exhibiting constriction of the VF.

Most of the studies dealing with VGB-attributed VF loss were retrospective, cross-sectional and analyzed the VF results in patients examined first after having started VGB therapy [8,16,21]. The advantage of our study is that it was prospective, performed using standardized method by the same examiner for five times during 2-years period. Quantitative assessment of the manual Goldmann kinetic VF is a very difficult task to perform. In previous studies with kinetic Goldmann perimetry I4e and V4e isopters [3] or III4e, and I2e isopters were used [8,19,22]. During manual kinetic perimetry isopters are drawn manually by the examiner, the area of isopters can be measured by planimetry but this procedure is complicated and time-consuming [3,4]. Other methods have also been used to quantitatively assess the results of manual Goldmann kinetic perimetry, such as the Esterman grid [4] and the mean radial degree (radius of isopters) [3,8,18,22]. The major advantage of SKP is that it is examiner-independent and enables constant speed of movement of the stimulus, automated measurement of area of isopters in deg^2^, moreover, it provides an electronic documentation of the results. SKP is also preferred by patients more than manual Goldmann kinetic perimetry. In the study comparing SKP with manual Goldmann perimetry in patients with advanced VF loss [12] a questionnaire was given to assess the preference of the patients. SKP was preferred by 52%, Goldmann was preferred by 32%, 16% had no preferrence. SKP was mostly preferred among patients with concentric constriction of the VF due to retinitis pigmentosa. In our opinion SKP can be recommended as a method of assessment of the visual function in the monitoring epilepsy patients taking VGB.

The number of patients in our study is relatively low (14), but the follow-up period was comparatively long - 24 months. Newman and colleagues examined one hundred patients at baseline, but after 18 months only 22 patients remained in the study [3]. In the study of Kinirons the mean follow-up time of the 41 patients was 2.2 years and the mean number of visual assessments was two [19]. Paul and co-workers observed 15 patients every 3 months for one year after 2-years period of VGB treatment [8]. In the most recent study regarding VAVFL 14 patients were monitored over a 10-year period with Goldmann manual kinetic perimetry (isopters I2e, I4e and I4e) [23]. In this study there was a high degree of variability observed in VF size between successive test sessions.

In the present study we found a significant decrease of the three isopters’ area during follow-up. Moreover, relationship was found between I2e isopters’ area and both the cumulative dose and the mean daily dose of VGB. Cumulative VGB dose has been already found the most significant predictor of the VF loss [4,23,24]. However, in the studies of Kalviainen [15], Kinirions [19] and Newman [3] no correlation was found between VF loss and either, the duration of VGB exposure or the cumulative dose. In the study performed by Conway and colleagues [25] maximum daily VGB dose was taken as an independent variable and was found as the most reliable indicator to exhibit VF defects. However, in this investigation only central VF was examined with automated static perimetry.

VF constriction was identified in 2 of 14 patients (14%), based on the assessment of two masked observers. The VF loss was mild according to the classification proposed by Wild and Kälviäinen [14,15] and in agreement with previous findings was more predominant nasally. Thus, it was not recognized by patients as the temporal and inferior fields are often more important for the function. Besch and colleagues have observed sparing of the temporal field in VGB patients using static perimetry [26]. Nasal predominance of the VF constriction was also observed by Midlefart in regard to static perimetry within 60 degrees of eccentricity [6] and by Daneshvar [27]. Kinirions and colleagues observed concentric constriction in 52.7% of the cohort [19], Newman in 20% of examined patients [3] but they used mean radial degree of III4e [19] or I4e [3] as an indicator. In our study the prevalence was low compared to that in the above-mentioned studies. In our study the serial VFs demonstrated inter-weaving of the isopters at baseline and throughout the subsequent studies, but there has already been reported a high variability of VF test in population of epilepsy patients taking VGB [19].

We have shown the usefulness of SKP in epilepsy patients. It has previously been shown that kinetic perimetry is better accepted by patients with neurological deficits than static perimetry [16]. Peripheral VF loss is clinically relevant during outdoor activity, as the peripheral retina is specialized in detecting moving objects. The position of isopters in manual kinetic perimetry is highly influenced by the individual RT of the patient [28]. RT is the time interval between the onset of the stimulus and the response. A longer RT shifts the isopter border towards the direction of stimulus movement. SKP gives advantage of measuring individual RT during single VF examination. The RT had not yet been investigated in patients taking VGB, it has been assessed so far in normal individuals and patients with advanced VF loss [5,10]. Taking into account other studies dealing with SKP, RT is prolonged in VGB patients comparing with normal individuals (370–756 ms) [10] and also in patients with advanced VF loss (794 ms) due to glaucoma, retinitis pigmentosa and hemianopia [29]. Prolongation of RT may additionally be explained by a decreased alertness in epilepsy patients. It has been shown that VGB treated patients can be impaired at detecting moving objects in the periphery, which may be caused by attention and recognition deficits. Slowing down of response times in participants treated by VGB has already been observed by Naili and co-workers [30]. Correcting the area of isopters to RT using SKP can eliminate the influence of patients’ fluctuating attention due to seizure activity and the drug treatment. In previous studies using Goldmann kinetic perimetry this criterion could have not been considered due to technological limitations. Delayed RT may mimic VF constriction under these conditions. Moreover, RT has been found to be the most important factor influencing the variability of the response and fatigue during SKP and can be used as a reliability indictor [31]. As in our study RT is prolonged in patients receiving VGB and there is correlation with decreasing isopters’area we can presume, that there may be high variability of response and fatigue in VGB patients performing SKP.

## Conclusions

In our study there was attenuation of area of III4e, I4e and I2e isopters during a period of 2 years. We found that RT, the cumulative dose and the mean daily dose of VGB influence isopters’ area obtained with SKP. We suggest performing VF testing prior to VGB treatment if patients are able to perform the test. Larger studies with SKP are necessary to explore the history and time course of the VAVFL.

## Abbreviations

VGB: Vigabatrin; VAVFL: Vigabatrin-attributable visual field loss; SKP: Semi-automated kinetic perimetry; RT: Reaction time; VF: Visual field; CBZ: Carbamazepin; CLZ: Clonazepam; OCZ: Oxcarbazepin; VPA: Valproid acid; LTG: Lamotriginum; SD: Standard deviation.

## Competing interests

The authors declare that they have no competing interests.

## Authors’ contributions

KN - design of the study, analysis and interpretation of the data, writing the manuscript, KR– acquisition of the data, MJ-statistical analysis, AB - contributed in the statistical analysis. KK, TŻ, MK, US-critical analysis of the results, revision of the manuscript; RM, PK and RR - acquisition and analysis of the data and revision of the manuscript for important intellectual content, general supervision. All authors read and approved the final manuscript.

## Pre-publication history

The pre-publication history for this paper can be accessed here:

http://www.biomedcentral.com/1471-2415/14/56/prepub
